# Effects of antirheumatic treatment with tocilizumab on longitudinal growth in children with juvenile idiopathic arthritis

**DOI:** 10.1186/1546-0096-12-S1-P62

**Published:** 2014-09-17

**Authors:** Ekaterina Alexeeva, Rina Denisova, Sanya Valieva, Tatyana Bzarova, Ksenya Isayeva, Tatyana Sleptsova, Evgeniya Chistyakova, Anna Fetisova, Olga Lomakina

**Affiliations:** 1 Rheumatology, Scientific Center of Children's Health of RAMS, Moscow, Russian Federation

## Introduction

Inflammation and glucocorticoid therapy are major factors in the growth retardation seen in children with severe forms of juvenile idiopathic arthritis (JIA).

## Objectives

The objective of the this study was to evaluate effects of antirheumatic treatment with recombinant humanised monoclonal antibody that acts as IL-6R antagonist (tocilizumab ) on longitudinal growth.

## Methods

Nineteen patients with systemic JIA was included into the study (8 boys and 11 girls). The mean age at first visit was 6.8 ± 2.4 years, and disease duration 4 (2,2;6) years. All patients had stage 1 of sexual development by Tanner scale and before therapy with tocilizumab had standard antirheumatic therapy. Anthropometric parameters were estimated one year before tocilizumab treatment, at the day of first infusion and in one year after tocilizumab treatment was started. SDS for height and height velocity calculated against the growth curves for European population (Auxology, Pfizer, Version 1.0).

Tocilizumab was administered intravenously once every 2 weeks at a dose of 8-12 mg/kg of body weight. In all patients who received corticosteroids before tocilizumab treatment dose of prednisolone was reduced from 0.5 (0.4; 0.6) to 0.1 (0.02; 0.2), but not in one case it was completely abolished. Treatment efficacy was assessed according to criteria ACR pedi scale.

## Results

Clinical response to the treatment was obtained in all patients included in this study. The ACR Pedi 50, 70 and 90 improvement were achieved by 3, 3, and 1 patients at Week 52, Inactive disease was achieved by 11 patients at week 52. The mean height SDS one year before treatment was -2.38 ±1.43 and -2.64±1.94 by the day of 1st tocilizumab infusion (p < 0.001). Height velocity SDS was -4.24 ±1.18 and -4.55 ±1.49 respectively (p < 0.001). After one year of treatment the mean height SDS was -2.27 ±1.85 and height velocity SDS 2.51 ±1.98 (p < 0.001).

## Conclusion

An intensified antirheumatic treatment with tocilizumab has a beneficial effect on growth in children with JIA. This effect might be related to the inhibitory effect of proinflammatory cytokines, especially Il-6, on the synthesis of IGF-1 and IGF-BP-3.

## Disclosure of interest

None declared.

